# Band ligation for a gastroesophageal junction Dieulafoy’s lesion

**DOI:** 10.11604/pamj.2017.26.181.9747

**Published:** 2017-03-29

**Authors:** Mohammed Amine Benatta, Jean Charles Grimaud

**Affiliations:** 1 Digestive Endoscopy Unit, Central Hospital of Army (HCA), Algiers, Algeria; 2 Gastroenterology Department, North Hospital, University of the Mediterranean, Marseille, France

**Keywords:** Band ligation, Dieulafoy´s lesion, gastroesophageal

## Image in medicine

In a 24 years old male presented with a first episode of massive hematemesis requiring resuscitation, a first upper endoscopic exam was inconclusive. The second endoscopic exam performed 24 hours later described a lesion as a little vessel with normal surrounding mucosa, without active bleeding (red arrow). This lesion was localized at the gastroesophageal junction (A) see the gastric mucosa (white arrow) and the esophageal mucosa (yellow arrow). This endoscopic finding of an isolated vessel without ulcer are characteristic of a Dieulafoy's lesion (DL). Given its particular localization and to prevent recurrence of bleeding, band ligation of this DL was found to be technically more suitable than haemoclip and/or injection. By Van Stiegmann technique using a Saeed Multi-Band ligator (Cook Medical) (B) the lesion with its surrounding mucosa were aspirated into the overtube (red arrow) and a single elastic band applied around the entire lesion (C). There were no immediate post procedure complications. Oral feeding was started 24 hours after and Intravenous Proton Pomp Inhibitor (PPI) therapy continued. The patient was discharged 48 h later with oral PPI. The course was uneventful and no recurrence of bleeding was reported after several months follow up. DL is rare and responsible in 0.3-6.7% of upper gastrointestinal bleeding but it is a potentially life-threatening condition. It is usually localized in the proximal stomach up to 6 cm from the gastroesophageal junction Endoscopic management is the gold standard for its diagnosis and treatment. Advances in endoscopy decreased the mortality of DL bleeding from 80% to 8.6%.

**Figure 1:**
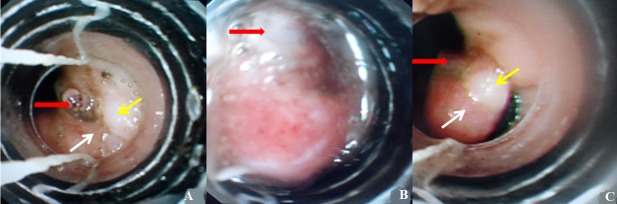
(A) little vessel with normal surrounding mucosa, without active bleeding (red arrow) localized at the gastroesophageal junction see the gastric mucosa (white arrow) and the esophageal mucosa (yellow arrow) corresponding to a Dieulafoy’s lesion; (B) the vessel (red arrow) with its surrounding mucosa were aspirated into the overtube; (C) a single elastic band applied around the entire lesion see the gastric mucosa (white arrow) , the esophageal mucosa (yellow arrow) and the vessel (red arrow)

